# Acceleration of Image Classification and Object Tracking by the Intel Neural Compute Stick 2 with Power Efficiency Evaluation on Raspberry Pi 4B

**DOI:** 10.3390/s25061794

**Published:** 2025-03-13

**Authors:** Tianyu Gao, Jozsef Suto

**Affiliations:** 1 Department of Informatics Systems and Networks, Faculty of Informatics, University of Debrecen, Kassai Street 26, 4028 Debrecen, Hungary; ethangaotianyu@mailbox.unideb.hu; 2 Department of IT, Eszterhazy Karoly Catholic University, Leanyka Street 4, 3300 Eger, Hungary

**Keywords:** Intel^®^ Neural Compute Stick 2, Raspberry Pi, OpenVINO™, object tracking, image recognition, power efficiency

## Abstract

This work investigates the efficiency and power consumption of using the Intel^®^ (Santa Clara, CA, USA) Neural Compute Stick 2 (NCS2) on the Raspberry Pi 4B platform to accelerate image classification and object tracking. The motivation behind this study is to enable the real-time operation of complex neural networks in embedded systems, potentially reducing the cost of deep learning neural network deployment and expanding industrial applications. This study also supplements the OpenVINO™ 2022.3.2 documentation by recording the application of the Raspberry Pi 4B combined with the NCS2 in the latest European software repositories. Supported by OpenVINO™ 2022.3.2 and the Deep SORT algorithm, this study consists of two distinct tests: image recognition and real-time object tracking. A single model is used for image recognition, while two models are deployed for object tracking. These test cases evaluate the performance of the execution hardware by varying the different number of models in different application scenarios and evaluating the impact of NCS2 acceleration under various conditions. The results indicate that, for the specific models used in this experiment, the NCS2 increases image recognition performance by approximately 400% and real-time object tracking by around 1400% to 1200%. The results presented in this work indicate that the NCS2 can achieve more than 50 FPS (frames per second) in image recognition and more than 20 FPS in object tracking. The power efficiency obtained by using the NCS2 can vary from 200% to 400%. These findings highlight the significant performance gains NCS2 offers in constrained hardware environments.

## 1. Introduction

Nowadays, the exploration of potential applications for deep learning models has become widespread. They are applied in areas such as autonomous vehicles, intelligent surveillance systems, and Internet of Things (IoT) devices [1,2,3]. These technologies have significantly increased productivity, but they have also introduced new challenges. As the efficiency of the models improves, their complexity and, consequently, their computational and memory requirements, also increase. This raises the demand for high-performance hardware, which leads to higher costs. Additionally, optimizing complex models requires a longer development time. At the same time, for such equipment, the long-term operation or simultaneous operation of multiple devices is expected, so from the perspective of embedded devices, energy consumption is also a major cost direction.

It is important to consider costs from two perspectives: purchasing costs and energy consumption costs. To reduce these expenses, the controllers of embedded systems (such as microcontrollers or single-board computers) are limited in computing capacity, physical memory, and consequently, energy consumption.

Deploying deep neural networks (DNNs) on IoT devices presents significant computational challenges due to their limited processing power, lack of dedicated accelerators, power constraints, and restricted numerical precision. Unlike high-performance desktop GPUs or cloud TPUs, IoT hardware is designed for energy efficiency and cost-effectiveness rather than intensive computation. These constraints directly affect the feasibility of real-time inference and necessitate optimization strategies to enable practical deployment.

One of the primary limitations stems from the low processing power of most IoT devices. As a result, running DNN inference on such hardware often leads to high latency, making real-time applications impractical. The execution of complex operations, such as convolutions and matrix multiplications, is considerably slower, particularly in scenarios requiring high-resolution image processing or rapid decision-making.

Furthermore, many IoT devices lack specialized hardware accelerators, such as GPUs, TPUs, or NPUs (neural processing units), which are essential for efficient deep learning inference. Without these accelerators, all computations must be executed on general-purpose CPUs, which are not optimized for parallel processing and deep learning workloads. This inefficiency further exacerbates power consumption and latency issues, making it difficult to achieve smooth, real-time inference. One of the contributions of this article is to accomplish the connection and use of TPUs in embedded system devices.

These limitations make it difficult to practically apply deep neural networks in embedded systems where real-time data processing is required [4]. Good examples of this would be object detection in autonomous vehicles [5,6]. A previous study [7] highlighted that the popular single-board computers such as the Raspberry Pi 3B+ and 4B, which are often used as control units in embedded systems, are not efficient enough to run well-known image classification models (e.g., SqueezeNet, MobileNetV2, MobileNetV3, EfficientNet), and their image processing speed does not even reach 1 FPS (frames per second). To address these limitations, major IT companies have developed accelerator modules. The most popular tensor processing units (TPUs) are probably the Intel (Santa Clara, CA, USA) Neural Compute Sticks (NCS1 and NSC2) [8]. These accelerators are designed to offload the workload from embedded system controllers and accelerate the decision-making process of artificial neural networks. Although the inference time of deep neural network (DNN) models on GPUs (graphical processing units) such as the Nvidia Xavier or RTX 2080 Ti GPUs will be lower than on the TPUs, TPUs can provide the maximum performance with the lowest energy consumption [9]. Therefore, they are well-suited to embedded systems.

The first version of the NCS was released commercially in 2017, while the second version became available at the end of 2018. Subsequently, several studies were conducted in which researchers started to use them in embedded system applications where image processing played the central role. For example, the authors of [10,11] used the NCS as part of an edge computing framework in a guide dog robot, while in [12], the NCS2 was used to accelerate the video processing of a threat detector system. Beyond the potential applications, an important question is as follows: how much do these devices increase the inference speed of DNNs compared to a single-board computer? After a detailed literature review, we found some studies where the authors examined the effectiveness of NCSs. Li et al. [13] compared the computation speed of a Raspberry Pi 3B+ with and without the NCS using well-known convolutional neural networks (CNNs) such as SqueezeNet, AlexNet, and GoogleNet. Their results showed 471%, 515%, and 647% inference speed increases on those models, respectively. Yepez et al. [14] tested their own two-stage decal recognition system on different hardware accelerators. Their results showed that the NCS2 increased the FPS rate of the system from 6.37 to 24.7 compared to a Raspberry Pi 4B. Mas et al. [15] achieved 11 FPS in a face recognition application (300 × 300 image size) on a Raspberry Pi 4B, which was extended with an NCS2. Arnautovic and Teskeredzic [16] evaluated the inference latency and energy consumption of the Raspberry Pi 3B+ with and without a NCS using the MobileNet SSDv2 model. Their results showed that the NCS decreased the inference time on a test image (its size is unknown) by 344%, while it increased the power consumption of the Pi from 6.834 W to 7.834 W. Nevertheless, the image processing speed is far behind real-time processing (~30 FPS), as the FPS value they achieved is only 4.51.

Unsurprisingly, the above studies indicate that NCS devices can deliver up to a 6-fold increase in FPS compared to a single-board computer such as the Raspberry Pi 4B, depending on the DNN model. However, the results of previous studies also showed that these systems, even with the help of the NCSs, were not capable of real-time image processing. In addition, doubts have also been raised regarding the efficiency of NCSs. For example, Cao et al. [17] claimed that the NCS does not bring significant inference latency improvements against a smartphone CPU (central processing unit) in the case of some well-known CNN modes such as SqueezeNet or MobileNetV2. On the other hand, the NCS2 increased the inference speed of those two modes by 533% and 243%. An additional issue we noticed is that the previously achieved results do not always align. As an example, the authors of [18] measured a 60 ms latency delay (16.67 FPS) using the YOLOv3 model on an image with 1200 × 900 pixels, while in the work of [19], the FPS of the same model was 2.5 on images with 1280 × 720 pixels.

Unlike previous articles, this study aims to highlight that real-time image processing can be achieved without constraints using the NCS2. Furthermore, this study also demonstrates that the NCS2 can perform a complex task such as object (person) tracking at a speed of over 20 FPS, even when the host is a single-board computer like the Raspberry Pi 4B. The main contributions of this work are the following:

- This research provides a comprehensive overview of how the NCS2 enhances the inference latency of DNN models, particularly for real-time applications running in resource-constrained hardware environments via two test cases (image recognition and object tracking). Our results indicate that the NCS2 can achieve more than 50 FPS in image recognition and more than 20 FPS in object tracking, which is a 1300–1500% speed increase compared to Raspberry Pi 4B.
- Since the host of the NCS2 can include different devices with varying clock frequencies, this study also examines the impact of changing the host machine’s clock frequency on the model’s inference speed behind image recognition and object tracking. To the best of our knowledge, this aspect has not been investigated in any previous study.
- This study thoroughly examines the Raspberry Pi 4B’s energy consumption with and without the NCS2 in both test cases and demonstrates that energy consumption is around 200-250% more efficient per image with the usage of the NCS2, while for person tracking, using the NCS2 led to approximately 400% power efficiency per frame.
- OpenVINO versions released after 2 March 2022 no longer support the NCS2. In order to ensure that the results presented in this study are reproducible, this article outlines how to set up the OpenVINO development environment on the Raspbian Stretch operating system. This is not a trivial task, as the most recent post about using the NCS2 with Raspberry Pi dates back four years, meaning that the available documentation is outdated and setting up the development environment poses challenges for developers.

## 2. Materials and Methods

### 2.1. Raspberry Pi Single-Board Computers

**Figure 1:**
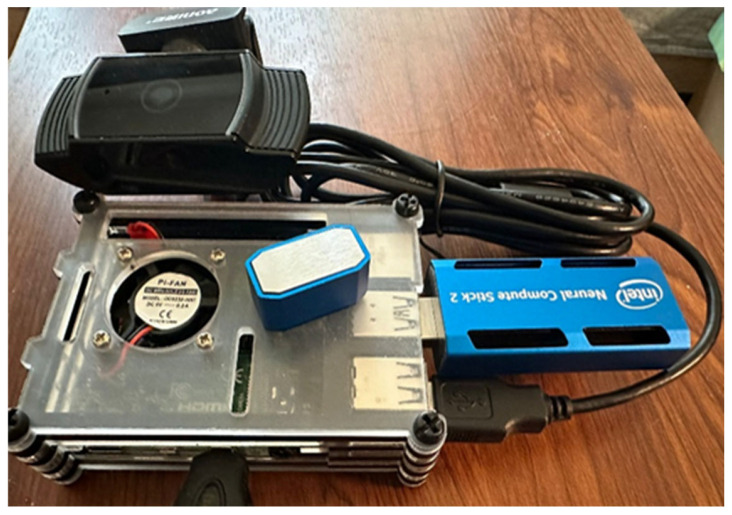
Used hardware components (Raspberry Pi 4B, NCS 2, USB camera).

The reason behind using Raspberry Pi (Cambridge, UK) 4B (Figure 1) lies in the main factors affecting the inference speed at a hardware level. The capability of parallel processing is important for speeding up the inference speed [19]. On a hardware level, having a GPU or multiple CPU cores can be the hardware requirement for parallelism. On the other hand, efficient data transfer can also speed up the neural network inference speed, such as a large memory bandwidth [20]. Regarding the efficient data transfer and large memory bandwidth, it is related to the RAM (random access memory) standard. We know that a computer that has a multi-core CPU, with a GPU and RAM that is large in bandwidth, can have much faster neural network inference speed. When examining the hardware specifications of Raspberry Pi candidate devices (3B+, 4B, 5), it is evident that with each new model, the hardware becomes more powerful. However, these improvements also lead to increased power consumption and cooling requirements.

Focusing on the RAM and CPU, a significant gap can be observed in performance between Raspberry Pi 4B and Raspberry Pi 3B. The difference between Raspberry Pi 4B and Raspberry Pi 5 is that the power consumption of Raspberry Pi 5 has been reduced by about 55% compared to Raspberry Pi 4B. Regarding the CPU, both the Raspberry Pi 4B and 5 have quad-core processors. However, the improvement of the Cortex-A76 architecture in Raspberry Pi 5 is the better architecture design, as stated by the official description. While clock speed can enhance performance, it is not the only factor influencing inference speed, and we can achieve a higher clock frequency by overclocking the Raspberry Pi 4B. The decision to use the Raspberry Pi 4B is based on its hardware improvements in CPU pipelining and memory bandwidth compared to the Raspberry Pi 3B. While the Raspberry Pi 5 offers better performance than the 4B, the difference in inference speed between the 4B and 5 is not as great as the leap from the 3B to the 4B. Additionally, the Raspberry Pi 5 comes at a higher cost and requires a dynamic cooling system, increasing the overall expense.

### 2.2. Intel Neural Compute Stick 2

A possible way to improve inference speed on single-board computers is to use hardware extensions such as NPUs (neural processing units) and VPUs (vision processing units). These accelerators improve inference efficiency by offloading the computationally intensive tasks from CPUs and processing the offloaded task using the dedicated hardware architecture [20]. In this work, the NCS2 has been used. An illustration of the stick can be seen in Figure 1. The NCS2 is a powerful hardware accelerator designed to improve inference speed for tasks such as image recognition and object tracking. The VPU is optimized to process visual data more efficiently than traditional CPUs and GPUs [21]. Vision or image processing places heavy demands on convolution calculations, which require many matrix calculations. This is particularly important for the two test cases that we implemented to measure the efficiency of the accelerator unit. The test cases are image recognition and object tracking. Both involve visual data processing.

The NCS2 processes image data much faster than a general-purpose processor, enabling us to achieve real-time performance. Given that both of our test cases rely heavily on visual data, the NCS2 architecture is well-suited for these tasks. In addition to performance, the low power consumption of the NCS2 makes it a good choice for embedded systems, where energy efficiency is an important consideration. The NCS2 usually operates with a power consumption range of 1 to 2.5 watts, depending on workload and usage conditions. This low power consumption is a key feature, enabling its use in power-constrained environments while still delivering substantial computing capabilities for neural network inferences. This makes it particularly suitable for edge devices, which are often limited by a restricted power supply. In many cases, ensuring that the hardware remained energy-efficient was crucial to the overall success of the project, as it aligned with the goal of building a system that can reliably operate in resource-constrained environments without sacrificing performance.

### 2.3. Installation of the Development Environment

The existing documentation for installing OpenVINO on Raspberry Pi faces numerous issues related to the software environment. It requires a complex build process for the OpenVINO Runtime, which can take several hours and often leads to errors such as version conflicts between modules, missing libraries, or even code-related problems that require manual intervention. These issues, along with the need to rebuild the software after each fix, present significant challenges and are time-consuming. As one of the outcomes of our work, we have created a GitHub 3.14 repository offering a simplified solution for installing the OpenVINO platform on Raspberry Pi through Linux shell scripts, while leveraging the latest European software repositories. The GitHub link is as follows: https://github.com/EthanGaoTianyu/NCS2Data/issues/1#issue 2611047696 (accessed on 10 March 2025).

We need to download the correct version of Python that is supported by OpenVINO 2022.3.2. This information is not available in the OpenVINO 2022.3.2 installation documentation, and it is only after initializing the OpenVINO environment that we will encounter an error related to the unsupported Python version. The supported Python versions are Python 3.6 to 3.10. In this work, Python 3.9.7 is chosen as the compatible Python version. Next, we need to install several essential development libraries for our Python environment. These libraries include libbz2-dev, libsqlite3-dev, libssl-dev, and libusb-1.0-0-dev. These development libraries are critical for subsequent Python compilations, ensuring that the compiled version of Python works properly and supports features such as encryption and databases.

OpenVINO can be separated into two main components: the OpenVINO Runtime and the OpenVINO Development Toolkit [22]. For our approach, we focus on installing only the inference part—the OpenVINO Runtime—on the Raspberry Pi. This allows us to run optimized models on the Raspberry Pi without the need for the full development environment. The more resource-intensive OpenVINO Development Toolkit, which includes model optimization and training tools, can be installed on a separate PC.

To ensure that OpenVINO uses the correct Python version for inference and other operations, we need to replace the symbolic links of Python3 and pip3 in the OpenVINO’s binary files with those in the Python 3.9 binary folder, by creating symbolic links pointing to Python3 and pip3, respectively. It is important to note that OpenVINO versions released after 2 March 2022 no longer support the NCS2.

Although parallelism can also be used for compiling OpenVINO, we do not recommend it, particularly on systems with limited memory, such as the Raspberry Pi 4B. Using parallelism can cause memory overhead, which may lead to a silent failure in the compilation process without generating error messages. On a Raspberry Pi 4B, the compilation process can take over five hours, and an unnoticed failure can result in significant wasted time and resources.

### 2.4. Image Classification

Image recognition is a process by which a machine uses neural networks to recognize objects, people, or patterns in an image. By using large amounts of labelled image data, deep learning models such as convolutional neural networks (CNNs) can learn to detect features and patterns that distinguish one object from another. Over time, the accuracy of these models improves, and increasingly complex images can be recognized. Image recognition is used in a wide range of applications such as facial recognition, autonomous driving, medical imaging, and object detection [23]. In this work, we will construct such an image recognition project using a MobileNetV3-Small architecture model.

MobileNetV3 is particularly well-suited for resource-constrained environments, such as embedded systems and mobile devices, offering reasonable image classification accuracy with minimal computational overhead. The model’s flexibility in adjusting floating-point precision and width multipliers allows it to balance performance and efficiency, making it highly adaptable across different application scenarios. MobileNetV3 is the third-generation architecture built based on MobileNetV1 and MobileNetV2. It primarily leverages the NAS (neural architecture search) technique, combining MobileNetV2′s features unique to MobileNetV3. MobileNetV3 provides two main variants, MobileNetV3-Large and MobileNetV3-Small. MobileNetV3-Small is designed to be lightweight, with reduced computational overhead, making it suitable for power-constrained devices like IoT devices or embedded hardware. In contrast, MobileNetV3-Large delivers higher accuracy but comes with increased computational demands, making it a better fit for medium-power devices such as mid-to-high-end smartphones or more capable edge devices [24]. Based on the MobileNetV3, we will use a pre-trained model provided by OpenVINO 2022.3.2, which is the v3-small_224_1.0_float model. The term “float” in v3-small_224_1.0_float refers to the data type of the model parameters being a floating-point number. The higher the precision, the more accurate the model becomes. Specifically, the model we are using supports 16-bit and 32-bit floating-point precision. In the OpenVINO documentation, they are referred to as “FP16” and “FP32”.

The width multiplier (*α*), set to 1.0, is a key hyperparameter that adjusts the complexity and computational demands of a model. It specifically determines the number of convolutional kernels in each layer of the network. A width multiplier of 1.0 signifies the use of the default number of convolutional kernels, representing the “standard width” of the model. Overall, MobileNetV3-Small provides an excellent trade-off between accuracy and computational cost, making it ideal for real-time, low-power devices, including resource-limited platforms like the Raspberry Pi.

### 2.5. Object Tracking

First, it is crucial to highlight the distinction between object detection and object tracking. Detection operates on individual frames, identifying objects in a single image without retaining any information from previous frames. Modern object detection models, such as YOLO, SSD, and Faster R-CNN, efficiently locate objects in each frame but do not establish temporal associations [25]. On the other hand, tracking extends detection by maintaining the identity of objects across multiple frames, enabling the system to follow them throughout the video sequence. Recent deep learning-based tracking methods, such as Deep SORT, Byte Track, and transformer-based trackers, leverage feature embeddings and association algorithms to improve identity preservation.

Object tracking is a challenging task due to several reasons. Firstly, environmental factors such as occlusions motion blur, and lighting variations lead to detection precision degradation [26]. In addition, due to the varying object size, the model needs to be scale-independent [27]. Beyond those challenges, attention must be paid to the edge computing platform’s energy consumption while running a deep learning-based model [28].

Beyond image recognition, our aim was to evaluate the computational efficiency of the NCS2 in handling a more complex deep learning task—object tracking. In this investigation, the tracked objects are people, and the goal is not only to detect them in each frame but also to track them across frames, ensuring the system correctly re-identifies individuals rather than simply drawing bounding boxes around detected objects in isolation. To achieve this, tracking algorithms must effectively combine object detection results with temporal association mechanisms while optimizing inference speed on resource-limited hardware.

**Figure 2:**
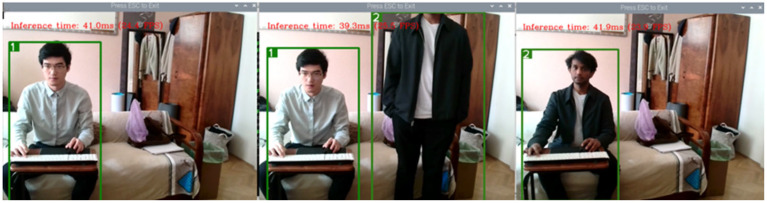
Illustration of person tracking with tree pictures taken one after the other.

An illustration of person tracking can be seen in Figure 2. In the left side picture of Figure 2, the first subject is marked with a green box (index 1). By this time, the relevant features of subject 1 are captured by the model to keep track of subject number 1. Subject 2 shows up in the next picture and is marked as index 2. Thereafter, he takes the place of subject 1 in the frame and is still marked as index 2. Even though subject 1 has left the picture, subject 2 will not be recognized as index 1. Such a process of update and association is the key to the tracking mechanism, while the detection of a subject will depend on the object detection model.

For detection, we use a pre-trained person-detection model from OpenVINO 2022.3.2, which is based on the MobileNetV2 architecture [29]. This architecture improves upon the original MobileNet by introducing depth-wise separable convolutions and inverted residuals, which reduce computational complexity while preserving high accuracy. MobileNetV2-based models are widely used in practical applications such as surveillance systems and autonomous vehicles, where real-time person detection on low-power devices is crucial due to the model’s efficiency and accuracy balance [30].

The person-reidentification-retail-0287 model provided by OpenVINO is designed for general-purpose person re-identification using OSNet (omni-scale network) architecture. This model takes a whole-body image as input and produces an embedding vector, which is essentially a numerical representation of the image’s key features. These embedding vectors can then be compared using cosine distance, a metric that measures the similarity between two vectors. If two images have similar vectors, it indicates that they are likely to belong to the same person [31]. OSNet is a deep learning architecture tailored for person re-identification (re-ID), which involves recognizing and matching individuals across different frames and camera views. Its key innovation is the omni-scale feature learning mechanism, allowing the network to capture and fuse features at multiple scales. This capability is essential for handling variations in appearance caused by factors like camera angles, lighting conditions, and environmental changes, making OSNet highly effective for robust re-identification tasks.

**Figure 3:**
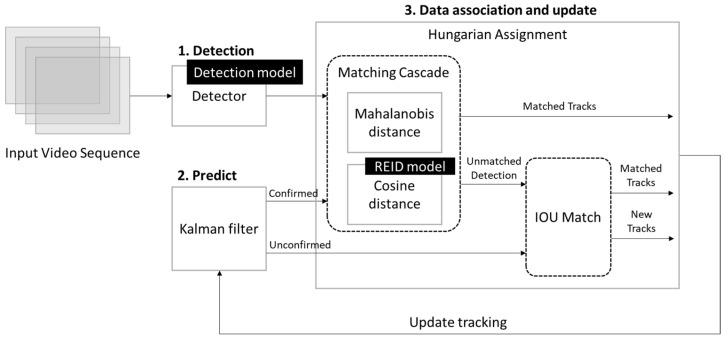
Deep SORT workflow chart [32].

Deep SORT (deep simple online and real-time tracking) is an improved version of the original SORT algorithm, designed for multi-object tracking. While SORT only uses motion information for tracking, Deep SORT enhances this by incorporating deep learning-based appearance features (Figure 3). This addition makes the algorithm more robust, especially in challenging scenarios like object occlusion and when dealing with objects that have similar appearances [20].

## 3. Results and Discussion

We have performed three different kinds of experiments to evaluate the improvement on both the speed and power efficiency of the NCS2 on the Raspberry Pi 4B platform.

### 3.1. Investigation of Inference Speed

**Figure 4:**
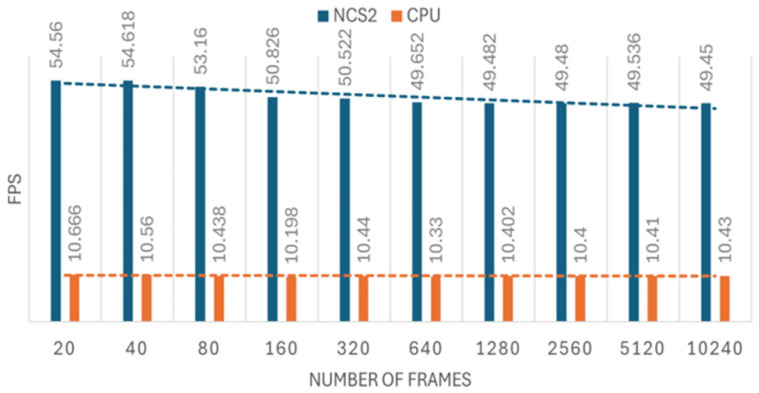
The speed of image recognition in FPS.

**Figure 5:**
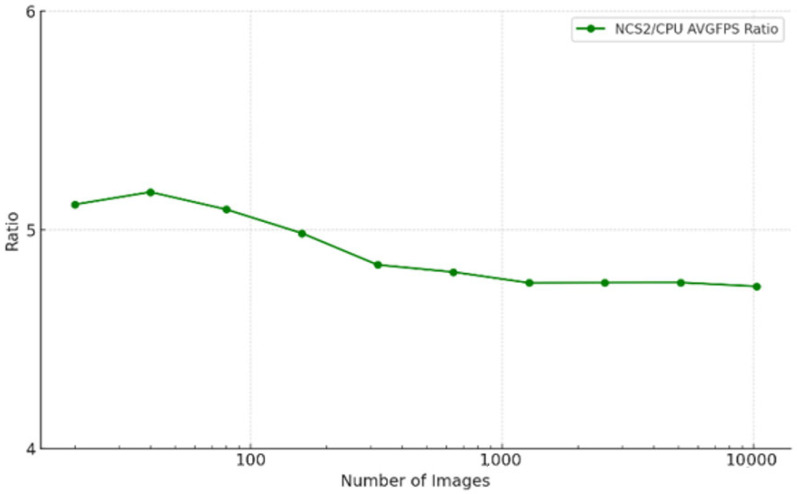
Efficiency improvement ratio of NCS2 against Raspberry Pi 4B in the image recognition test case.

The Myriad X VPU has dedicated hardware accelerators that efficiently run CNN-based tasks, making it especially suitable for edge devices with limited computing resources such as Raspberry Pi. The NCS2 excels at CNNs and is good at handling common deep convolution operations. In this work, two projects were developed to test the efficiency improvement of the NCS2 inference process for different types of CNNs in image recognition and real-time object tracking tasks. For image recognition, we have created a loop and set a timer to test the model’s performance on different numbers of images. By running the image recognition model on the CPU of the Raspberry Pi 4B (CPU) and NCS2, we can compare the FPS values to quantify the speed increase that is obtained using the NCS2. This approach allows us to analyze the efficiency of NCS2 processing different workloads, thereby gaining insight into how hardware acceleration affects the inference speed of real-time deep learning tasks. When running inference performance tests, it is best to perform pre-inference to ensure that the device is loaded and in a stable state. Devices such as the NCS2 may have an additional initialization overhead when running a model for the first time, which can affect the first inference time. We can avoid such an overhead by first performing an inference operation that is not counted in the test. The experiments are conducted with 10 sets in which each set contains different numbers of images starting from 20 to 10240. The test images were self-produced, and their size was adjusted to match the image dimension expected by the model, which is 224 × 224 pixels. The number of images in the sets comes from the following formula: 10 * 2^(set number)^. Each set is conducted five times for both CPU and MYRIAD to ensure accuracy. The result can be seen in Figure 4 and Figure 5.

**Figure 6:**
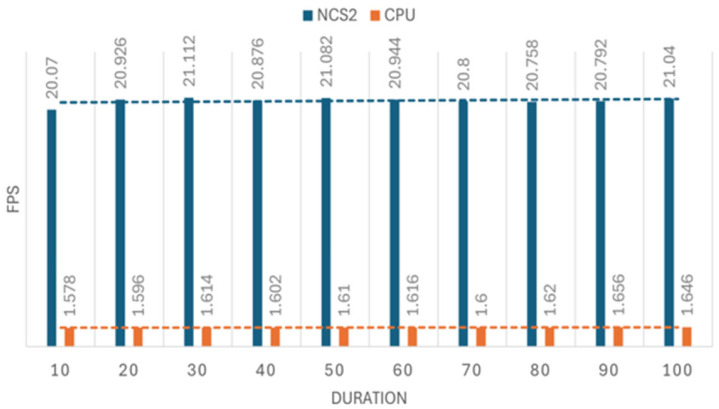
The speed of person tracking in FPS.

**Figure 7:**
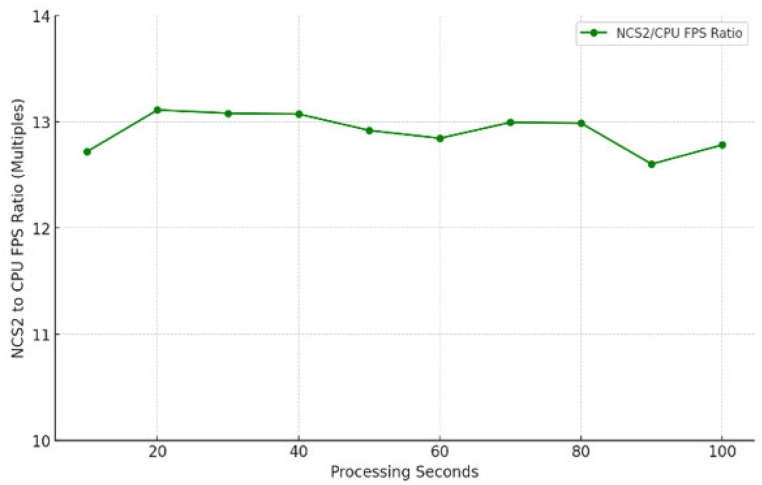
Efficiency improvement ratio of NCS2 against Raspberry Pi 4B in the person tracking test case.

In object tracking, we employed person-detection for detecting individuals in each frame and person-reidentification for tracking and maintaining identity consistency across frames. The experiment involved 10 sets of data with different durations of video streams from a USB camera, ranging from 10 to 100 s. The time duration for each set is determined according to the following formula: time amount = set number * 10. During the experiment, inference times were recorded, from which the frames per second were calculated, and saved to a text file for further analysis. Additionally, the total number of frames processed during each time interval were also documented to determine the average FPS. Each set of tests is repeated five times to ensure the reliability of the measurement. The results of this investigation can be seen in Figure 6 and Figure 7.

As shown in Figure 7, the ratio is approximately 13. However, when the test is conducted without the frame counter (code lines used to track the number of processed frames) and FPS documentation (code lines used to record the current FPS), the improvement can reach up to 15× (equivalent to a 1400% increase). Additionally, removing the frame counter and FPS documentation allows the FPS of using the NCS2 to increase from 20 to 27. These findings highlight that code robustness and efficiency can significantly affect inference speed and overall performance. As we can see from Figure 6, the FPS of using the host device (CPU) alone is very low, around 1.6, and for the NCS2, the FPS reached around 21, which is very close to 30 FPS.

### 3.2. Overclock and Downclocked Performance Experiment

As we know, one way to improve CPU performance is by increasing its clock frequency, which in turn may improve inference speed. On the Raspberry Pi, we can modify system files to either overclock or downclock the CPU. In this experiment, we adjusted the Raspberry Pi 4B CPU clock frequency to three levels: the default 1.7 GHz, an overclocked 2.0 GHz, and a downclocked 1.5 GHz. The objective was to investigate how the CPU frequency influences inference speed.

**Figure 8:**
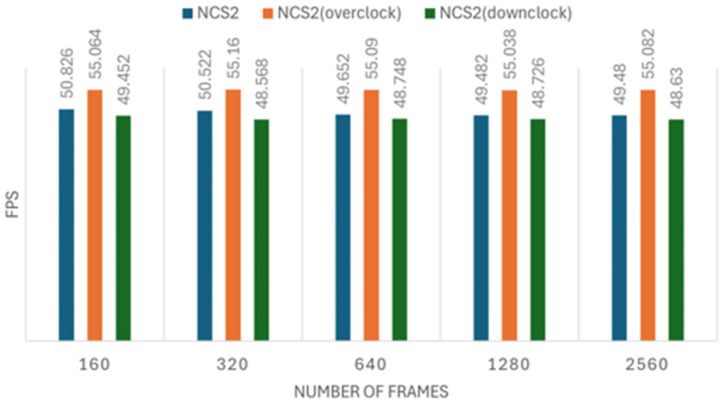
The effect of clock frequency changes on image recognition speed when using the NCS2.

**Figure 9:**
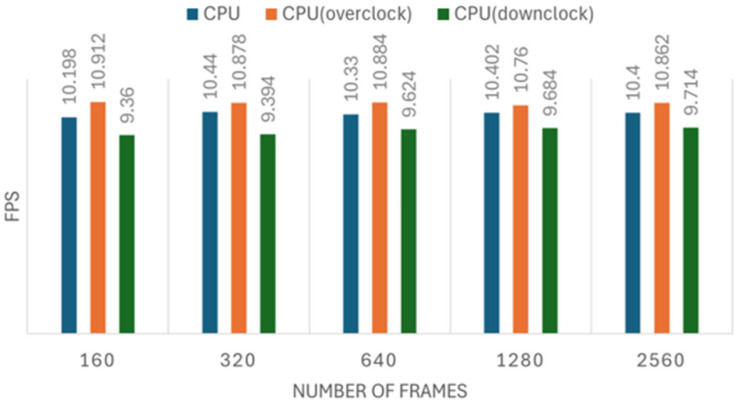
The effect of clock frequency changes on image recognition speed when using the CPU.

**Figure 10:**
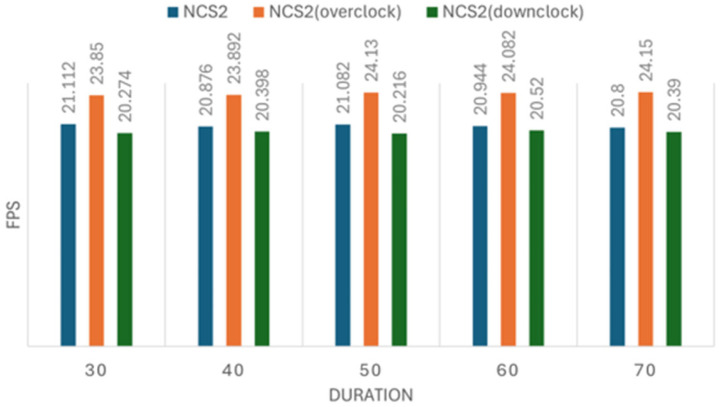
The effect of clock frequency changes on object tracking speed using the NCS2.

**Figure 11:**
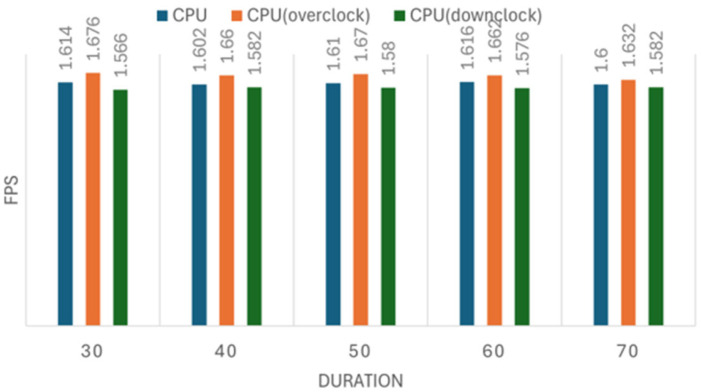
The effect of clock frequency changes on object tracking speed using the CPU.

We conducted five sets of experiments for both the image recognition and person tracking tasks, each including three configurations: normal, overclocked, and down-clocked. The image recognition experiment processed datasets of 160, 320, 640, 1280, and 2560 images per set, while the tracking experiment followed a similar setup. Each measurement was repeated five times to ensure reliability. The results of these experiments are presented in Figure 8, Figure 9, Figure 10 and Figure 11. Figure 8, Figure 9, Figure 10 and Figure 11 illustrate the impact of CPU clock frequency adjustments on inference speed.

The results indicate that changes in CPU frequency affect performance in both the CPU and NCS2 test sets for object tracking and image recognition. This suggests that increasing the CPU frequency can enhance inference speed for both devices to some extent. However, the improvement is limited; raising the CPU clock from 1.5 GHz to 2.0 GHz results in only a modest increase in FPS. This implies that the CPU-based inference faces inherent performance constraints.

Moreover, when compared to the performance gains achieved with the NCS2 accelerator, the effect of overclocking appears minimally. For instance, while an overclocked CPU at 2.0 GHz provided only a slight FPS increase, the NCS2 acceleration resulted in several-fold improvements in inference speed. These results reinforce the conclusion that CPU overclocking alone is not an optimal solution for accelerating deep learning inference, especially in resource-constrained environments.

### 3.3. Power Efficiency

When designing embedded systems, it is important to understand the system’s energy consumption. Since energy consumption varies depending on the computational load, accurate measurements are required to precisely estimate the energy needed to complete a predefined task. It is well-known that the Raspberry Pi devices can be powered via GPIO (general purpose input output) pins. Therefore, we first attempted to monitor the system’s energy consumption in the two test cases using an INA260 module [15]. However, this was not particularly stable. Especially when we connected the NCS2 and the camera to the Raspberry Pi, the device always ran out of voltage, even when we boosted the voltage by an extra 20%. Our experience shows that this issue can be avoided when powering the Raspberry Pi 4B using USB-C. In this case, the Raspberry Pi 4B can run perfectly at high power. To record the voltage and current per second used by the Raspberry Pi 4B, an OWON ODP3122 power meter has been used in combination with a split 3A USB type-c cable to connect the Raspberry Pi to the power meter.

**Figure 12:**
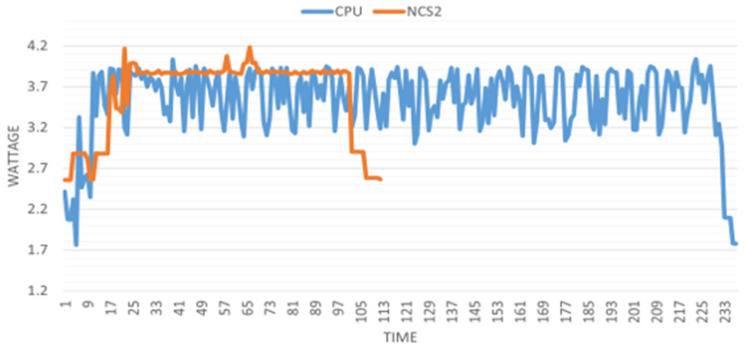
Power consumption of the Raspberry Pi 4B with and without the NCS2 during image recognition.

**Figure 13:**
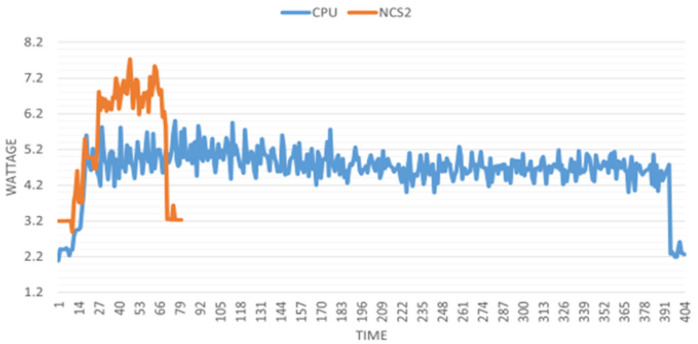
Power consumption of the Raspberry Pi 4B with and without the NCS2 during person tracking.

This investigation involves data collection from the two projects: image recognition and person tracking. For image recognition, datasets of 1000, 2000, and 5000 frames are processed, while for person tracking, datasets of 200, 500, and 1000 frames are used. Each dataset is tested three times to ensure reliability. A 10 s buffer time is allocated before and after each process to account for setup and stabilization. For illustration, the power consumption (in wattage) of the system can be seen in Figure 12 and Figure 13 during image recognition (with 2000 images) and person tracking (with 500 frames).

**Figure 14:**
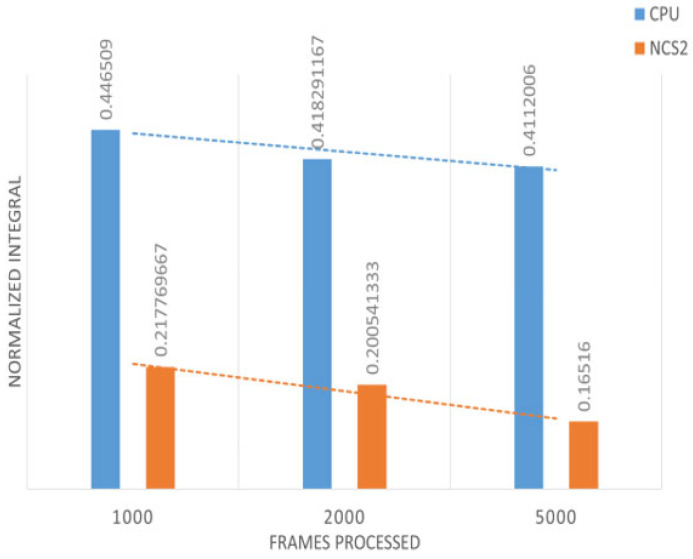
Power efficiency in the case of image recognition.

**Figure 15:**
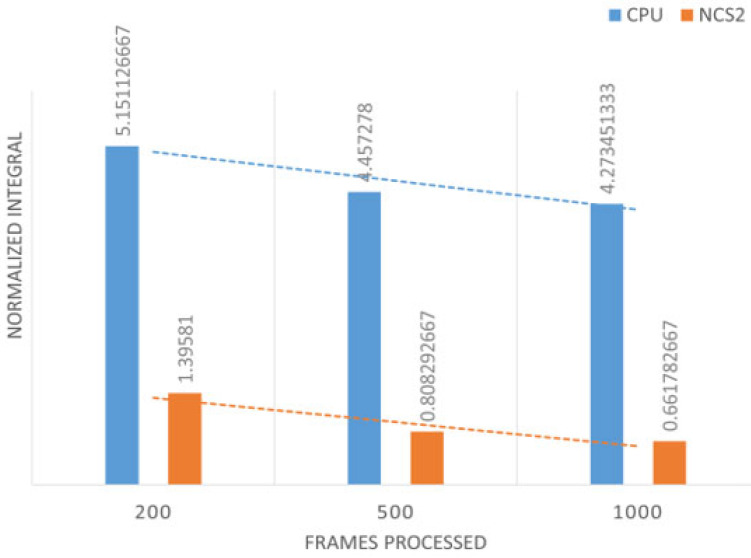
Power efficiency in the case of person tracking.

The above figures clearly show that although the NCS2 increased the overall energy consumption, it significantly reduced the model’s inference time. The general power increase is 0.3 W when the NCS2 is plugged in but not in use, and 2 W to 2.5 W when actively running inference. To better display all the collected power consumption data in an integrated manner, we introduced a bar plot where the y axis values of the above figures have been summed up and divided by their total duration (Figure 14 and Figure 15). This gives the average energy consumption for a fixed number of frames processed in the corresponding group. We take the average of the data obtained in this way with the data from the other two power measurements in each group. In this case, the smaller value means the higher power efficiency.

Figure 14 and Figure 15 provide several key insights. First, as the number of frames processed increases, the efficiency of both the host device and NCS2 improves. This is because certain preparatory steps, such as compilation, decoding, and related subprocesses, are executed in a nearly fixed time interval before actual inference begins. As a result, when the overall execution time increases, these fixed overheads become less significant relative to the total computation, leading to better power efficiency. From the power efficiency experiments, we can see that with the same number of tasks, the NCS2 is much more power-efficient than the host device (CPU) due to its improvement in the inference speed. For image recognition, it is around two to three times more power-efficient than the host device, while for person tracking, it is up to four times more power-efficient per frame.

## 4. Conclusions

The experiments of this work show that the Intel Neural Compute Stick 2 (NCS2) significantly boosts performance in DNN-based tasks, offering a 400% increase in image recognition and up to 1200% to 1400% in person tracking. This significant performance improvement is attributed to the NCS2′s ability to efficiently utilize its dedicated computational resources in performing convolutional operations and optimizing the inference process, thus reducing the overall inference time of the system.

At the same time, the results of this work also confirm that the effect of increasing the inference speed only by increasing the CPU frequency is relatively limited, especially in application scenarios involving deep learning models, where traditional CPU architectures exhibit certain bottlenecks when facing parallel computing demands. These findings suggest that in industrial applications and academic research where the goal is to maximize inference speed, priority should be given to investing in dedicated neural network accelerators such as the NCS2, rather than relying solely on increasing CPU performance.

Moreover, the NCS2 is more power-efficient, consuming up to four times less power in tracking tasks and approximately two times less in image recognition, making it well-suited for real-time applications in resource-constrained edge computing environments. The efficiency and affordability of the NCS2 are particularly suited to edge computing environments, enabling real-time inference capabilities even on resource-constrained devices, thus providing a future for DNN-controlled embedded systems and IoT applications.

## Data Availability

The test images used in this study are available on the following GitHub repository: https://github.com/EthanGaoTianyu/NCS2Data/issues/1#issue 2611047696 (accessed on 10 March 2025).
